# Copy number variation analysis in 189 Romanian patients with global developmental delay/intellectual disability

**DOI:** 10.1186/s13052-022-01397-1

**Published:** 2022-12-30

**Authors:** Diana Miclea, Sergiu Osan, Simona Bucerzan, Delia Stefan, Radu Popp, Monica Mager, Maria Puiu, Cristian Zimbru, Adela Chirita-Emandi, Camelia Alkhzouz

**Affiliations:** 1 Department of Molecular Sciences, “Iuliu Hatieganu” University of Medicine and Pharmacy, Pasteur Street, No 6, 400349 Cluj-Napoca, Romania; 2 Emergency Clinical Hospital for Children, Pasteur Street, No 6, 400349 Cluj-Napoca, Romania; 3 Department of Mother and Child, “Iuliu Hatieganu” University of Medicine and Pharmacy, Cluj-Napoca, Romania; 4 Department of Neuroscience, “Iuliu Hatieganu” University of Medicine and Pharmacy, Cluj-Napoca, Romania; 5 “Victor Babes” University of Medicine and Pharmacy, Timisoara, Romania; 6 Polytechnic University, Timisoara, Romania

**Keywords:** Global developmental delay, Intellectual disability, Chromosomal microarray analysis, Copy number variants, Etiology

## Abstract

**Background:**

Developmental delay and intellectual disability represent a common pathology in general population, involving about 3% of the pediatric age population, the genetic etiology being often involved. The aim of this study was to determine the clinically relevant copy number variants in patients diagnosed with global developmental delay/intellectual disability in our population, using the chromosomal microarray analysis.

**Methods:**

We analyzed 189 patients diagnosed with global developmental delay/intellectual disability, presented in Clinical Emergency Hospital for Children, Cluj-Napoca. The patients were completely clinically investigated, including dysmorphic and internal malformations evaluation, psychiatric, neuropsychological and metabolic evaluation, standard karyotyping. Genomic analysis was done using chromosomal microarray analysis.

**Results:**

Pathogenic findings (including uniparental disomy) and variants of unknown significance were detected in 53 of 189 patients (28.04%). Pathogenic copy number variants and uniparental disomy were observed in 35 of 189 patients (18.51%). Two patients presented uniparental disomy for chromosome 15, one with clinical phenotype of Prader-Willi syndrome and the other with clinical phenotype with Angelman syndrome. Within the category of pathogenic findings, the recurrent copy number variants were seen in 21 of 35 patients (60%).

**Conclusions:**

The increased percentage of pathogenic structural variants observed in patients with global developmental delay/intellectual disability analyzed by chromosomal microarray technique supports its use in patients with a non-specific phenotype such as these neurodevelopmental disorders. The high percentage of recurrent pathogenic variants between these findings is a finding that support their initial evaluation when a genetic testing algorithm could be a useful option.

## Background

Developmental delay and intellectual disability represent a common pathology, affecting 1–3% of children, the etiology being represented by genetic factors in more than a half of these patients [1–3]. Global developmental delay (GDD) is a diagnosis reserved for a child under five years, being defined as a significant delay, under two standard deviations (SD), in two or more developmental domains (gross or fine motor abilities, speech/language, cognition, social/personal and activities of daily living) [4]. Intellectual disability (ID) is a diagnosis established beginning with the age of five years, when the following three criteria are met simultaneously: defective intellectual function (usually measured by intellectual coefficient), defective adaptative function (conceptual, social, or practical skills) and onset of these deficits during the developmental period [5]. Not all the patients with GDD diagnosis will fulfill the criteria for ID diagnosis after the age of five years.

With advanced genomic technologies, as chromosomal microarray analysis (CMA) and exome/genome sequencing, the genetic etiology in GDD/ID is now identified in more than 50% of these patients [3, 5].

The G-bands karyotype identified numerical or structural chromosomal abnormalities in approximately 5% of GDD/ID patients (in some studies, up to 15% of cases) [5–8], 21 trisomy being the most frequently seen (in about 70% of these patients) [6, 8]. Recurrent microdeletions/microduplications (mainly involving 22q11.2, 7q11.23, 17p11.2, 15q11–13, 16p11.2, 1q21.1 and other regions) are observed in about 5% of cases, usually being identified by Fluorescent In Situ Hybridization (FISH), Multiplex Ligation-dependent Probe Amplification (MLPA) or quantitative Polymerase Chain Reaction (qPCR) techniques [8, 9]. A first-tier test in genetic investigations in GDD/ID is now represented by CMA, due to an important diagnostic yield, of 15–25% in patients with GDD/ID [10–13], preferred over G-bands karyotype, FISH, MLPA or qPCR techniques, due to a higher sensitivity and better genomic resolution for copy number variants (CNVs) detection [10].

Pathogenic single nucleotide variants (SNVs) or indels variants, in monogenic or oligogenic disorders, are identified by exome/genome sequencing in 15–30% of GDD/ID patients, tests usually performed after a negative CMA analysis [10, 12–15].

The other unexplained causes in GDD/ID patients could be related to environmental teratogens (including the fetal alcohol exposure, valproate exposure or infections), perinatal factors (prematurity, asphyxia, or other neonatal complications) or postnatal causes (as CNS infections, traumatisms, toxic, psychosocial environment).

The aim of this study was to determine the clinically relevant CNVs in Romanian children diagnosed with GDD/ID, using Single Nucleotide Polymorphism (SNP) array technology.

## Methods

We analyzed 189 patients diagnosed with GDD/ID, presented in Clinical Emergency Hospital for Children Cluj-Napoca, between January 1st 2015 and July 1st 2017. The age of the patients was between 1 and 18 years. The inclusion criteria was the diagnosis of GDD or ID. An exclusion criteria was the presence of 21 trisomy confirmed by karyotype. GDD/ID diagnosis was based on the intelligence quotient evaluated by Wechsler Intelligence Scale for Children test (WISC-IV) and development quotient (for children younger than 6 years), evaluated by Portage test and A Developmental NEuroPSYchological Assessment test (NEPSY). The patients were completely clinically investigated, including dysmorphological evaluation, internal malformations evaluation, psychiatric and neuropsychological examinations, metabolic evaluation, standard karyotyping. Brain imaging and electroencephalogram (EEG) were indicated by the neurologist. Other investigations was performed depending on clinical indication of each patient.

The research was approved by Ethics Committee of Clinical Emergency Hospital for Children, Cluj-Napoca. Written informed consent was obtained from the parents of all the participants in the study.

### High density SNP array analysis

The deoxyribonucleic acid (DNA) was purified by Wizard® Genomic DNA Purification Kit (Promega, Madison, WI, USA), using 3 ml peripheral blood, sample collected for each patient. Then, a SNP array analysis was done using Infinium OmniExpress-24 BeadChip array kit (Illumina, San Diego, CA, USA) and the platform iScan System (Illumina, San Diego, CA, USA). The SNP array kit allowed the analysis of about 700,000 markers. For bioinformatic analysis it was use the Genome Studio software version 2.0 (Illumina, San Diego, CA, USA). The interpretation of each CNV was done using the recommendations of American College of Medical Genetics [16, 17].

## Results

The study group included 189 patients, 91 girls (48.14%) and 98 boys (51.85%), with an age between three and 18 years (Table 1). The average age was 11.17 years and 28 of 189 patients (14.81%) were five years old and under the age of five years (with the GDD diagnosis), the others 161 patients (85.19%) were older (with ID diagnosis). Pathogenic findings (including pathogenic CNVs and uniparental disomy - UPD) and variants of unknown significance (VOUS) were detected in 53 of 189 patients (28.04%). Pathogenic CNVs and UPD were observed in 35 of 189 patients (18.51%). Clinical characteristics are described in Table 1.

**Table 1:** Clinical characteristics in patients with GDD/ID

Clinical features	***n*** (%)
**Gender**	*189 patients*
Male	98 (51%)
Female	91 (48%)
**Age**	*189 patients*
< or = 5 years	28 (14%)
> 5 years	161 (85%)
**CNVs**	*189 patients*
Pathogenic	33 (17.4%)
Uniparental disomy	2 (1%)
VOUS	18 (9.5%)
**Clinical features in patients with pathogenic CNVs**	*33 patients*
Dysmorphic features	27 (81%)
Short stature	2 (6%)
Obesity	6 (18%)
GDD/ID	33 (100%)
Microcephaly	1 (3%)
Epilepsy	4 (12%)
Autism spectrum disorders	1 (3%)
Hypotonia	1 (3%)
Language delay	3 (9%)
Associated internal malformation	7 (21%)

The pathogenic CNVs detected in our patients are described in Table 2. Two patients presented UPD for chromosome 15, one with clinical phenotype of Prader-Willi syndrome and the other with clinical phenotype of Angelman syndrome (patients 76 and 78). Among pathogenic CNVs, 22 patients (66.7%) presented deletions and 11 patients (33.3%) had duplications.

**Table 2:** Pathogenic CNVs observed in our GDD/ID patients

Patient	CNV (del/dup)	Chr	Start (hg19)	Stop (hg19)	Size (Kb)	Known Genetic Syndrome	Patient phenotype
**1**	Del	17q12	34,856,055	36,248,918	1392	17q12 deletion syndrome	GDD/ID, dysmorphic features, ataxia
**3**	Dup	Xq27.1-q27.3	139,283,418	146,699,586	7416	*SOX3* deletion	GDD/ID
		22q11.1-q11.21	17,397,498	18,984,519	1587	Cat Eye syndrome	
**5**	Dup	1q41-q44	219,786,897	249,212,668	29,425	1q41–44 duplication	GDD/ID, dysmorphic signs
**6**	Dup	16p13.13-p13.2	8,226,775	12,071,213	3844	16p13.2 deletion syndrome	GDD/ID, ASD, epilepsy, obesity
**45**	Del	14q32.2	99,448,000	100,800,103	1352	14q32 deletion syndrome	GDD/ID, short stature, dysmorphic features
**55**	Del	5q35.2-5q35.3	175,346,223	177,484,097	2137	Sotos syndrome	Sotos syndrome, GDD/ID, dysmorphic features, language delay, obesity, CNS and renal malformation
**59**	Del	1q21.2–21.2	146,501,348	147,911,246	1409	1q21.1 deletion syndrome	GDD/ID, dysmorphic features
**61**	Dup	16p11.2	28,615,243	29,028,905	413	16p11.2 duplication syndrome	GDD/ID, dysmorphic features
**62**	Dup	18p11.32–11.21	112,535	14,791,236	18,678	18p Deletion syndrome	GDD/ID, dysmorphic features
**66**	Dup	17p11.2	16,777,177	20,239,827	3462	Potocki-Lupski syndrome	GDD/ID, dysmorphic features, obesity
**67**	Del	22q11.21	18,886,915	21,462,353	2575	DiGeorge syndrome	GDD/ID, obesity, dysmorphic features
**71**	Del	6q15q21	91,305,608	111,699,368	20,393	6q syndrome deletion	GDD/ID, dysmorphic features
**90**	Del	4p16.1-p16.3	71,566	8,357,645	8286	4p deletion syndrome	Wolf-Hirschhorn syndrome
**91**	Del	16p11.2	29,595,483	30,187,676	592	16p11.2 deletion syndrome	GDD/ID, language delay, dysmorphic syndrome, obesity
**106**	Del	9p24.3-p13.1	46,587	39,179,289	39,132	9p deletion syndrome	GDD/ID, dysmorphic syndrome
**109**	Dup	16p24.3	89,542,695	89,656,251	113	16q24.3 deletion syndrome	GDD/ID, dysmorphic features
**117**	Del	18p11.32–11.31	13,034	4,390,081	4377	18p Deletion syndrome	GDD/ID, dysmorphic features
**118**	Del	15q11.2-q31.1	23,656,946	28,535,266	4878	Prader-Willi syndrome	GDD, hypotonia
**130**	Del	15q11.2-q31.1	23,656,946	28,535,266	4878	Prader-Willi Syndrome	GDD/ID, obesity
**136**	Del	1p36.33-1p36.32	82,154	3,821,782	3739	1p36 deletion syndrome	GDD/ID, dysmorphic syndrome
**149**	Dup	16p11.2	29,595,483	30,215,621	620	16p11.2 duplication syndrome	GDD/ID, short stature, deafness
**150**	Del	7q11.23	73,110,603	73,702,525	592	Williams syndrome	GDD/ID, dysmorphic syndrome
**151**	Del	Xp22.31	6,456,940	8,135,053	1678	Xp22.3 microdeletion syndrome	GDD/ID, dysmorphic syndrome
**153**	Del	7p15.3p21.1	18,814,931	23,539,546	4726	Partial monosomy 7p	GDD/ID, dysmorphic syndrome
**154**	Del	17q21.31	44,163,925	44,177,103	13	17q21.31 deletion syndrome (KANSL1 – exon 3)	GDD/ID, dysmorphic features, cardiac and genitourinary malformation
**156**	Dup	16p12.2-p11.2	21,610,804	30,198,151	8587	16p11.2–p12.2 duplication syndrome	GDD/ID, dysmorphic features
**157**	Dup	18p11.21–11.32	13,034	15,375,878	15,362	18p Deletion syndrome	GDD/ID, epilepsy
**160**	Del	16p11.2	28,593,316	28,995,057	401	16p11.2 deletion syndrome	GDD/ID, dysmorphic features
**161**	Del	22q11.21	18,889,490	21,797,812	2908	DiGeorge syndrome	GDD/ID, dysmorphic features, cardiac and renal malformation
**165**	Del	1q21.1	145,394,955	145,755,813	360	1q21.1 deletion syndrome	GDD/ID, epilepsy, dysmorphic features, forearm agenesis
**166**	Del	4q22.2-4q24	94,543,233	107,486,817	12,943	4q deletion syndrome	GDD/ID, dysmorphic syndrome, language delay
**184**	Del	4p16.2–16.3	48,283	5,405,805	5357	4p deletion syndrome	GDD/ID, epilepsy, cardiac malformation, dysmorphic features
**189**	Dup	18q21.2–23	48,866,388	77,888,708	29,022	18q21q24 duplication	GDD/ID, microcephaly, epilepsy, dysmorphic features

*CNV* copy number variant, *del* deletion, *dup* duplication, *chr* chromosome, *kb* kilobase, *GDD* global developmental delay, *ID* intellectual disability, *ASD* autism spectrum disorder

Recurrent pathogenic CNVs were observed in 21 of 35 patients (60%) with pathogenic findings, thus: 15q11.2-q31.1 deletion (two patients), 4p16 deletion (two patients), 22q11.21 deletion (two patients), 22q11.21 duplication (one patient), 16p11.2 proximal deletion (two patients), 16p11.2 proximal duplication (one patient), 18p11 duplications (two patients), 18p11 deletion (one patient), 7p11.23 deletion (one patient), 5q35 deletion (one patient),1q21 deletion (two patient), 1p36 deletion (one patient), 17p11.2 duplication (one patient), 17q21.31 deletion (one patient), Xp22.31 deletion (one patient) (Table 2). The clinical phenotype was suggestive for the etiological diagnosis in four of 189 patients (2.11%) and confirmed by SNP array analysis, thus: Wolf-Hirschhorn syndrome (4p16 deletion), Williams syndrome (7p11.23 deletion), Sotos syndrome (5q35 deletion) and Prader-Willi syndrome (15q11.2-q31.1 deletion). For most patients, the clinical phenotype was not suggestive for a particular etiology.

The patients observed with VOUS in our study group are described in Table 3.

**Table 3:** VOUS observed in our GDD/ID patients

Patient	CNV/UPD	Chromosome	Start	Stop	Size (Kb)	Major genes involved	Patient phenotype
3	Dup	3q26.1	161,577,780	166,471,417	4893	BCHE, SI	GDD/ID
	Dup	4q28.2-4q28.3	130,609,436	138,430,265	7820	PCDH10, PABPC4L	
	Dup	4q12q13.2	58,771,770	67,055,049	8283	EPHA5, LPHN3, TECRL	
5	Del	11q25	133,531,291	134,868,407	1337	JAM3, ACAD8, NCAPD3	GDD/ID, dysmorphic features
8	Dup	17q21.33	48,263,589	48,607,252	344	COL1A1,XYLT2	GDD/ID, autism spectrum disorder
62	Dup	21p11.1	34,097,891	34,853,011	755	IFNAR2, PARK20	GDD/ID, dysmorphic features
65	Dup	15q12	26,874,395	26,888,344	14	GABR3	GDD/ID
68	Del	10q21	68,107,483	68,150,124	42	CTNNA3	GDD/ID, dysmorphic features
84	Del	12p12.1	23,836,212	23,840,513	4.3	SOX5	GDD/ID, dysmorphic features, hypotonia
85	Del	1q34	237,584,925	237,597,163	12	RYR2	GDD/ID, dysmorphic features, deafness, mitral insufficiency
	Del	16q22.1	70,513,384	70,519,783	6	COG4	
110	Dup	11q13.4	70,969,719	71,419,408	449	DHCR7	GDD/ID, obesity
123	Del	19p13.11	33,882,222	33,893,008	10	PEPD	GDD/ID, dysmorphic features, spastic paraplegia
	Del homozygous	7p22.1	4,823,971	4,839,265	15	AP5Z1	
163	Dup	3q27.1	184,010,230	184,038,969	28	PARK18	GDD/ID, autism spectrum disorder, language delay
164	Del	6p25.1	5,256,116	5,391,419	135	FARS2, LYRM4	GDD/ID, obesity, hypospadias, language delay
173	Dup	22q11.21	18,877,787	19,008,108	130	DGCR5, DGCR6, DGCR9, PRODH	GDD/ID, ataxia
178	Dup	22q11.21	18,895,227	19,008,108	112	DGCR5, DGCR6, DGCR9, PRODH	GDD/ID, dysmorphic features
183	Del	10q22.3	79,313,729	79,331,919	18	KCNMA1	GDD/ID, West syndrome, ataxia
185	Del	18q21.1	43,655,010	43,743,081	88	ATP5A1	GDD/ID, microcephaly, dysmorphic features, short stature
186	Del	Xp11.4	38,230,704	38,246,882	16	OTC	GDD/ID, obesity, cryptorchidism
188	Del	Xp11.4	38,235,792	38,256,737	20	OTC	GDD/ID, dysmorphic features

*CNV* copy number variant, *UPD* uniparental disomy, *kb* kilobase

## Discussions

In this study group of Romanian patients with GDD/ID we identified pathogenic CNVs, UPD or VOUS in 28% of patients. Pathogenic CNVs and UPD were seen in 18.5% of patients. The recurrent pathogenic CNVs were seen in 60% of patients with pathogenic findings (CNVs or UPD).

A similar percentage of pathogenic findings analyzing patients with GDD/ID was also seen in other studies [18–25], supporting the important diagnosis yield given by this analysis, indicated as first-tier test in GDD/ID [10]. A genomic approach for the patients with an unspecific phenotype such as isolated or syndromic GDD/ID is useful, in our research, the clinical etiological diagnosis was indicated in only 2% of cases, similar with other study [9].

Recurrent CNVs were identified in 60% of pathologic findings, the same percentage being observed by other study [26], these CNVs having in some cases a potential recognizable phenotype, even if quite variable in some patients, compared to the classical clinical picture. This could be an argument to continue giving an importance to the phenotype evaluation, which could bring a diagnosis in some patients, that can be confirmed more easily and less expensive by MLPA technique. The same recurrent CNVs seen in our study, described above, were also noted by other studies [26, 27]. Chromosome 18 was often involved in pathogenic CNVs, four patients presenting large deletion/duplication: 18q21.2-q23 duplication, 18p11.32-p11.21 duplication and 18p11.32-p11.21 deletion.

Some patients presented some very rare and particular CNVs, which will be described below. The patient 3, a 12-year-old boy with isolated GDD/ID, presented as a particularity a pathogenic 22q11.1-q11.21 duplication of 1.5 Mb (cat eye syndrome) associated to a pathogenic Xq27.1-q27.3 duplication of 7.4 Mb duplication, the last one including more OMIM genes, *SOX3* being a known morbid OMIM gene, coding for a transcription factor implicated in neurodevelopment, which is associated with X-linked intellectual disability and panhypopituitarism or growth hormone deficiency. These features were described for other patients in literature, our patient presenting isolated GDD/ID without endocrine or other features [28–31]. The patient 5, a 18-year-old girl with GDD/ID and dysmorphic signs, presented 29.4 Mb duplication of 1q41-1q44 region, which included 43 morbid OMIM genes (including *ZBTB18*), a similar CNV being described in other patients, most of them also presenting short stature or associated internal malformations [32–35], features not observed in our patient.

In patient 6, a 11-year-old boy with GDD/ID, epilepsy, autism spectrum disorder (ASD) and obesity was detected a 16p13.2-16p13.13 duplication (3.8 Mb), including *GRIN2A* gene -known to be associated with epilepsy and GDD/ID- and also 16p13.2 region -known to be associated with 16p13.2 duplication syndrome - *USP7* gene usually involving ASD and GDD/ID- these features were also described in our patients [27]. The pathogenic CNVs described in patient 45 – a 3-year-old girl with GDD/ID, short stature and dysmorphic features - is a 14q32.2 deletion (1.3 Mb), which included genes involved in ID, as *YY1* gene, responsible of Gabriela de Vries syndrome [36], overlapping CNVs were described in Decipher patients (260,834, 291,402), with similar phenotypes as our patient, the cases with this CNV are very rare. 6q15-q21 deletion of 20.3 Mb seen in patient 71 is another rare CNV already noted in association with GDD/ID [37–39], including an important number of OMIM genes involved in neurodevelopment. In patient 153, presenting with GDD/ID and dysmorphic features, was observed the 7p15.3-p21.1 deletion (4.7 Mb), also described in association with ID [40], for this patient it is interesting that *TWIST1* gene, associated with Saetre-Chotzen syndrome, is also included in this deletion, being responsible for dysmorphic features presented in our patient. The deletion in 4q22.2-q24 region in patient 166, who presents GDD/ID, dysmorphic features and language delay is also a very rare CNVs, it was described in patients with similar features [41, 42].

## Conclusion

The pathogenic findings, as pathogenic CNVs or UPD, were observed in 18.5% patients, thus supporting the use of chromosomal microarray technique in patients with a non-specific phenotype such as GDD/ID. Recurrent CNVs were observed in 60% patients of those with pathogenic findings, as: 15q11.2-q31.1 deletion, 4p16 deletion, 22q11.21 deletion, 22q11.2 duplication, 16p11.2 deletion, 16p11.2 duplication, 18p11 duplications, 18p11 deletion, 7p11.23 deletion, 5q35 deletion,1q21 deletion, 1p36 deletion, 17p11.2 duplication, 17q21.31 deletion, Xp22.31 deletion.

## Data Availability

Relevant data generated or analyzed during this study are included in this published article. The other datasets used and/or analyzed during the current study are available from the corresponding author on reasonable request.

## Abbreviations

GDD: Global developmental delay
SD: Standard deviation
ID: Intellectual disability
CMA: Chromosomal microarray analysis
FISH: Fluorescent in situ hybridization
MLPA: Multiplex ligation-dependent probe amplification
qPCR: Quantitative polymerase chain reaction
CNVs: Copy number variants
SNVs: Single nucleotide variants
Indels: Insertion and/or deletion of nucleotides into genomic DNA (deoxyribonucleic acid), less than 1 kb in lenght
CNS: Central nervous system
SNP array: Single nucleotide polymorphism array
WISC-IV: Wechsler Intelligence Scale for Children
NEPSY: Developmental NEuroPSYchological Assessment
EEG: Electroencephalogram
DNA: Deoxyribonucleic acid
UPD: Uniparental disomy
VOUS: Variant of unknown significance
Del: Deletion
Dup: Duplication
Chr: Chromosome
Kb: Kilobase
ASD: Autism spectrum disorder
Mb: Megabase
